# Responsible use of large language models in manuscript authorship, peer review, and editorial processes: a Delphi consensus among editors-in-chief of anaesthesia and pain medicine journals (RULE-AP)

**DOI:** 10.1016/j.bja.2026.01.029

**Published:** 2026-02-26

**Authors:** Alessandro De Cassai, Burhan Dost, John G. Augoustides, Leonard Azamfirei, Zekeriyya Alanoğlu, Liana Maria Azi, José Andrés Calvache, Vladimir Cerny, Stefan De Hert, Abdelazeem Eldawlatly, Michaela K. Farber, Diogo Sobreira-Fernandes, Michael R. Fettiplace, Dario Galante, Rakesh Garg, Heidi V. Goldstein, Alfredo Abad-Gurumeta, Lalit Gupta, Hugh C. Hemmings, Christopher A. Jones, Marc C. Hochberg, Joel D. Katz, Hyun Kang, Gül Köknel Talu, Durval C. Kraychete, Ruth Landau, Sangseok Lee, Hillary D. Lum, Christina Lundgren, Thamasi Rekha Makuloluwa, Paolo Martelletti, Tonya M. Palermo, Philip J. Peyton, Pierrick Poisbeau, James P. Rathmell, Antoine Roquilly, Stephan K.W. Schwarz, Madhuragauri Shevade, Paul A. Sloan, BobbieJean Sweitzer, Ary Serpa Neto, Philip F. Stahel, Zerrin Özköse Şatırlar, Alparslan Turan, Dennis C. Turk, Massimiliano Valeriani, Mads U. Werner, Paul J. Young, Igor Borisovich Zabolotskikh, Kai Zacharowski, Szymon Zdanowski

**Affiliations:** 1 Department of Medicine (DIMED), University of Padua, Padua, Italy; 2 Institute of Anesthesia and Intensive Care, University Hospital of Padua, Padua, Italy; 3 Department of Anesthesiology and Reanimation, Ondokuz Mayis University Faculty of Medicine, Samsun, Turkey; 4 Department of Anesthesiology and Critical Care, Perelman School of Medicine, University of Pennsylvania, Philadelphia, PA, USA; 5 Department of Anaesthesiology and Intensive Care, George Emil Palade University of Medicine, Pharmacy, Science, and Technology of Targu Mures, Targu Mures, Romania; 6 Department of Anesthesiology, School of Medicine, Washington University in St. Louis, St. Louis, MO, USA; 7 School of Medicine of Bahia, Federal University of Bahia (UFBA), Salvador, Brazil; 8 Department of Anesthesiology, Universidad del Cauca, Popayan, Colombia; 9 Department of Anesthesiology, Erasmus MC, Rotterdam, The Netherlands; 10 Department of Anesthesiology, Perioperative Medicine and Intensive Care, Masaryk Hospital and J.E. Purkyne University, Usti nad Labem, Czech Republic; 11 Charles University in Prague, Faculty of Medicine in Hradec Kralove, Czech Republic; 12 Department of Anesthesia, Pain Management and Perioperative Medicine, Dalhousie University, Halifax, NS, Canada; 13 Department of Basic and Applied Medical Sciences, Faculty of Medicine and Health Sciences, Ghent University, Ghent, Belgium; 14 Department of Anesthesia, College of Medicine, Assiut University, Asyut, Egypt; 15 Department of Anesthesia, College of Medicine, King Saud University, Riyadh, Saudi Arabia; 16 Department of Anesthesiology, Mass General Brigham, Boston, MA, USA; 17 Harvard Medical School, Boston, MA, USA; 18 Department of Anaesthesiology, Luz Arrábida Hospital, Vila Nova de Gaia, Portugal; 19 Department of Anesthesiology, University of Illinois at Chicago, Chicago, IL, USA; 20 Department of Anesthesia, Intensive Care and Pain Therapy, Hospital ‘G. Tatarella, Cerignola’, ASL Foggia, Italy; 21 Department of Onco-Anaesthesia and Palliative Medicine, Dr B.R.A. Institute Rotary Cancer Hospital and National Cancer Institute, All India Institute of Medical Sciences, New Delhi, India; 22 Department of Anesthesiology, North Florida/South Georgia Veterans Health System, Gainesville, FL, USA; 23 Department of Anesthesiology, University of Florida College of Medicine, Gainesville, FL, USA; 24 Department of Anesthesiology and Critical Care, Hospital Universitario Infanta Leonor, Madrid, Spain; 25 Department of Pharmacology, Medicine, Universidad Complutense, Madrid, Spain; 26 Department of Anaesthesiology and Intensive Care, Maulana Azad Medical College, New Delhi, India; 27 Department of Anesthesiology, Weill Cornell Medicine, New York, NY, USA; 28 Division of Geriatrics and Palliative Care, Duke University School of Medicine, Durham, NC, USA; 29 Department of Epidemiology and Public Health, University of Maryland School of Medicine, Baltimore, MD, USA; 30 Department of Medicine, University of Maryland School of Medicine, Baltimore, MD, USA; 31 Department of Psychology, York University, Toronto, ON, Canada; 32 Department of Anesthesiology and Pain Medicine, College of Medicine, Chung-Ang University, Seoul, Korea; 33 Department of Pain Medicine, Medical School of Istanbul University, Istanbul, Turkey; 34 Universidade Federal da Bahia, Postgraduate Program in Medicine and Public Health, Salvador, BA, Brazil; 35 Department of Anesthesia, Columbia University Irving Medical Center, New York, NY, USA; 36 Department of Anesthesiology and Pain Medicine, Sanggye Paik Hospital, Inje University College of Medicine, Seoul, Korea; 37 Division of Geriatric Medicine, Department of Medicine, University of Colorado School of Medicine, Aurora, CO, USA; 38 Wits Donald Gordon Medical Centre and University of the Witwatersrand, Johannesburg, South Africa; 39 Department of Clinical Sciences, Faculty of Medicine, General Sir John Kotelawala Defence University (KDU), Ratmalana, Sri Lanka; 40 School of Health, Unitelma Sapienza University of Rome, Rome, Italy; 41 Department of Anesthesiology & Pain Medicine, University of Washington School of Medicine, Seattle, WA, USA; 42 Center for Child Health, Behavior & Development, Seattle Children's Research Institute, Seattle, WA, USA; 43 Department of Anaesthesia, Austin Health, Melbourne, Victoria, Australia; 44 Department of Critical Care, University of Melbourne, Parkville, Victoria, Australia; 45 Cognitive and Adaptive Neuroscience Laboratory, Centre National de la Recherche Scientifique, Université de Strasbourg, Strasbourg, France; 46 Center for Research in Transplantation and Translational Immunology, Nantes Université, CHU Nantes, INSERM, Nantes, France; 47 Service d’Anesthesie Réanimation, Immuno-infectiologie, Nantes Université, CHU Nantes, INSERM, Nantes, France; 48 Department of Anesthesiology, Pharmacology & Therapeutics, The University of British Columbia, Vancouver, BC, Canada; 49 Department of Anesthesia, St. Paul’s Hospital, Providence Health Care, Vancouver, BC, Canada; 50 Department of Training Programs, Chest Research and Training Pvt Ltd, Pune, India; 51 Department of Anesthesiology, University of Kentucky, Lexington, KY, USA; 52 University of Virginia, Charlottesville, VA, USA; 53 Australian and New Zealand Intensive Care Research Centre (ANZIC-RC), School of Public Health and Preventive Medicine, Monash University, Melbourne, Victoria, Australia; 54 Department of Intensive Care, Austin Hospital, Melbourne, Victoria, Australia; 55 Department of Critical Care, Melbourne Medical School, University of Melbourne, Austin Hospital, Melbourne, Victoria, Australia; 56 Department of Critical Care Medicine, Hospital Israelita Albert Einstein, Sao Paulo, Brazil; 57 Department of Surgery, Brody School of Medicine, East Carolina University, Greenville, NC, USA; 58 Department of Specialty Medicine, Rocky Vista University College of Osteopathic Medicine, Parker, CO, USA; 59 Department of Anesthesiology and Reanimation, Faculty of Medicine, Gazi University, Ankara, Turkey; 60 Center for Outcomes Research, Department of Anesthesiology and Pain Medicine, University of Texas, Houston, TX, USA; 61 Developmental Neurology Unit, Ospedale Bambino Gesù, Rome, Italy; 62 Systems Medicine Department, Tor Vergata University of Rome, Rome, Italy; 63 Multidisciplinary Pain Center 7612, Department of Anesthesia, Pain and Respiratory Support, Neuroscience Center, Rigshospitalet, Copenhagen, Denmark; 64 Intensive Care Unit, Wellington Hospital, Wellington, New Zealand; 65 Medical Research Institute of New Zealand, Wellington, New Zealand; 66 Department of Critical Care, University of Melbourne, Melbourne, Victoria, Australia; 67 Department of Anesthesiology, Intensive Care Medicine and Transfusiology, Kuban State Medical University, Krasnodar, Russia; 68 Department of Anaesthesiology, Intensive Care and Pain Therapy, University Hospital Frankfurt, Goethe University Frankfurt, Frankfurt, Germany; 69 Department of Anaesthesiology and Intensive Therapy, Medical University of Gdansk, Gdansk, Poland

**Keywords:** anaesthesia and pain medicine journals, Delphi consensus, editorial policy, large language models, research integrity, responsible artificial intelligence, scientific publishing ethics

## Abstract

This article presents a Delphi consensus developed by a panel of editors-in-chief of anaesthesiology and pain medicine journals to guide the responsible use of large language models (LLMs) in academic publishing. LLMs offer potential benefits for scientific writing, including language editing, summarisation, translation, information organisation, and support for non-native English speakers, but their misuse raises concerns about accuracy, transparency, confidentiality, and research integrity. Through a three-round modified Delphi process involving 53 editors-in-chief or their delegates, 59 statements were generated and categorised into guidance for authors, editors, reviewers, and publishers with a particular attention to LLM disclosure practices and perceived risks. The consensus recognises that LLMs are useful tools in academic publishing for authors, reviewers, and editors. However, their use must be guided by ethics, legality, and principles of transparency and accountability. LLMs may assist with limited editorial and authorial tasks provided that their use is fully disclosed and all outputs are verified by humans. The consensus also emphasises the inappropriateness of using LLMs to generate original or ideative content, which should remain a strictly human responsibility. Moreover, LLMs must not generate data, references, conclusions, or entire manuscripts, nor be used for editorial decisions or peer-review reports. Editors expressed concerns about ‘hallucinations’, erosion of critical skills, confidentiality breaches, and the proliferation of low-quality LLM-generated manuscripts. The resulting guidance highlights transparency, human accountability, and careful verification as essential principles for integrating LLMs into scholarly workflows while preserving the integrity of scientific publishing.

**Editor’s key points**

• Large language models can support selected aspects of scientific writing and editorial workflows.
• Permitted use does not diminish manuscript quality, provided that it is transparently disclosed, carefully verified by humans, and remains under full human accountability.
• Because large language model technology is rapidly evolving, these recommendations should be regarded as a living framework that requires regular updates.

Large language models (LLMs) are advanced deep learning architectures, typically based on transformer neural networks and natural language processing techniques, and trained on vast corpora of text data to learn statistical patterns and semantic relationships within languages. These models are capable of performing a wide range of language-related tasks, including comprehension, generation, translation, summarisation, and reasoning, without task-specific supervision.^1^

This technology has rapidly entered daily life and has permeated the scientific community as it can be used for a wide range of academic purposes, including scientific writing, literature and database searching, information organisation, initial draft generation, and proofreading.^2^ Some recent studies have demonstrated that LLMs can significantly enhance the English academic writing proficiency of non-native speakers.^3^ They can reduce cognitive load by automating routine tasks, allowing researchers to focus on higher-order reasoning and analysis.^4^ LLMs provide real-time feedback that improves structure, clarity, and coherence in academic texts.^5^

However, several concerns have been raised regarding the indiscriminate use of LLMs in academic scholarship. It has been clearly established that these models can generate false facts (often referred to as ‘hallucinations’)—such as fabricated data, stereotyped imagery, non-existent references, or ‘tortured’ language, by which we mean syntactically fluent but semantically distorted phrasing that obscures meaning or misrepresents concepts. Although such language is certainly not unique to LLM-generated text and is also encountered in human-authored manuscripts, its automated and large-scale production poses a particular threat to the academic integrity if left undetected. Their use for scientific writing has been likened to taking a shortcut that produces faster but potentially less accurate academic work.^6^ Moreover, if authors do not disclose the use of LLMs, distinguishing between human- and machine-generated text becomes a significant challenge,^7^ even when using currently available LLM detection tools.^8^

All members of the scientific ecosystem—authors, reviewers, editors, and publishers—have encountered this novel tool and face the urgent need to identify its possible applications, benefits, limitations, and the ethical implications associated with its use.^9^ Moreover, patients, as the ultimate beneficiaries of research, are directly affected when studies are poorly designed, conducted, or reported. Therefore, fostering a transparent and responsible discussion about LLM use is of paramount importance.

Given the growing ubiquity of these tools in academic workflows, and the need to balance the concerns associated with their use and the potential benefits of responsible application, the present manuscript reports a Delphi consensus among 50 editors-in-chief (EICs) of anaesthesiology and pain medicine journals. The aim of this consensus is to provide a practical framework for authors, editors, reviewers, and publishers on how LLMs can be safely and effectively integrated into academic processes while preserving the integrity of scientific publication.

## Methods

The present manuscript has been reported in accordance with the CREDES (Conducting and REporting DElphi Studies) checklist available for consultation as Supplementary material 1.^10^

### Composition of the project administrators and working group

The two administrators of the project (ADC and BD) were responsible for the overall conduct of the Delphi process, including the identification of the work group and the formulation of the initial survey questions.

The working group was identified as editors holding an active EIC role in journals categorised under ‘Anesthesiology’ or ‘Pain Medicine’ in *Scopus* or *Clarivate Journal Citation Reports* at the time of recruitment.

EICs were identified through the editorial board pages of each eligible journal. Contact information was retrieved primarily from the journal website. If unavailable or not working, the corresponding author’s e-mail listed in recent publications by the EIC was used as an alternative. After the initial invitation, participants received up to two reminder e-mails, sent at intervals of at least 1 week. EICs were given the option to personally participate or to nominate a delegate to participate on their behalf.

### Modified Delphi process

The Delphi method was selected because it enables a structured and iterative collection of expert opinions in areas where empirical data are limited and policy development relies on informed judgement. A consensus-based approach is considered the most appropriate to capture diverse expert perspectives while minimising the influence of dominant individuals and reducing potential geographic or institutional bias.^11^

The study comprised three mandatory rounds (one qualitative and two quantitative rating rounds), with an optional fourth round for validation. All procedures were conducted online using LimeSurvey (LimeSurvey GmbH, Hamburg, Germany). Participants’ responses were anonymised to ensure that each contributor’s opinion was equally weighted to minimise bias.

The first round, developed by the project initiators (ADC and BD), aimed to explore participants’ perceptions of opportunities, risks, and policy priorities related to the use of LLMs in manuscript preparation, peer review, and editorial workflows. Participants responded to a series of open-ended questions (Supplementary material 1) covering general perspectives, ethics and authorship, acceptable use, disclosure and transparency, and additional considerations. There were no restrictions on the number or scope of issues participants could raise, and no predefined statements were provided. Two independent coders (ADC and BD) performed an inductive thematic analysis of all Round 1 responses following a standard multi-step approach: familiarisation, initial coding, theme development, codebook generation, and coder reconciliation.^12^ Themes derived from participant responses were then translated into draft candidate consensus statements that reflected the full range of views expressed by the panel. These draft statements were further reviewed by a third independent reviewer (MF) who was blinded to the work of the previous coders to ensure that each item was formulated as a concise declarative proposition suitable for rating. These statements were organised into conceptual domains derived from the qualitative analysis. The finalised list of items was reviewed for clarity, consistency, and redundancy before being advanced to Round 2.

In Round 2, participants rated each statement on a 9-point Likert scale (1=not important/inappropriate; 9=very important/appropriate) and were offered the opportunity to provide optional comments or suggest rewording. For each item, we calculated the median, 25–75% interquartile range (IQR), and the proportion of respondents rating 7–9. Items meeting predefined consensus criteria were either accepted with strong consensus, accepted, rejected, or carried forward.

In Round 3, participants received the list of items ‘approved with strong consensus’; the list of items ‘accepted without strong consensus’, modified with minor rephrasing based on suggestions from the working group; the list of items without consensus, revised according to the feedback and input provided by the working group; and the list of rejected items. Participants were invited to re-rate items that had no strong consensus or no consensus using the same 9-point scale. Items with strong consensus or those that had been rejected were not re-voted.

An optional Round 4 (validation) was planned if more than five to eight important items remained borderline or if participants requested additional deliberation.

Results from all the rounds are available as Supplementary material 1, and in accordance with the FAIR principles, the anonymised voting data for all rounds are provided in Supplementary material 2.^13^

### Consensus definitions

Consensus thresholds were defined *a priori* as follows: strong consensus (median ≥8, IQR ≤1.5, and ≥80% of participants rating 7–9); consensus (median ≥7, IQR ≤2.0, and ≥70% rating 7–9); and consensus against (median ≤3, IQR ≤2.0, and ≥70% rating 1–3). Items not meeting these criteria were classified as showing no consensus. Items that showed consensus against in Rounds 2 and 3, and items that failed to reach the consensus threshold by the final round, were rejected. It should therefore be noted that consensus and strong consensus reflect the collective assessment of the working group rather than unanimity, and that some participants may not have endorsed a statement even when strong consensus was reached.

### Ethical, statistical, and sample size considerations

The RULE-AP Delphi project was approved by the Clinical Research Ethics Committee of Ondokuz Mayis University (approval number 2025/456). Informed consent was obtained from each participant through explicit checkbox confirmation at the beginning of the first-round survey, after a detailed explanation of the project’s aims and objectives. The protocol and the data from each round were available for consultation for the working group at Open Science Framework (reference: osf.it/973vf, first registered on August 28, 2025).

All responses were anonymised before analysis. Identifiers such as journal name or participant name were stored separately and were accessible only to the project administrators (ADC and BD). Quantitative data were analysed descriptively using medians, IQRs, and percentage distributions.

The sample size was determined *a priori*. The study was designed to proceed only if at least 20% of eligible journals agreed to participate. Given that 147 journals met the inclusion criteria, the minimum required number of consenting editors was set at 29.

## Results

### Working group

A total of 147 journals were identified as eligible for inclusion (Supplementary material 1). After the initial invitation and subsequent reminders, 53 EICs from 53 journals (36.0%) agreed to participate in the project—one journal was represented by two EICs, and two journals shared the same EIC. Two EICs from two journals (1.4%) declined participation, and no feedback was received from the remaining 92 journals (62.5%). Among the 53 participating EICs, eight delegated a member of their editorial board to take part in the project on their behalf (see Supplementary material 1 for a full list of the included members and their corresponding roles).

The workgroup’s journals included *A & A Practice*, *Acta Anaesthesiologica Belgica*, *Agri*, *Anaesthesia and Intensive Care*, *Anaesthesia Critical Care & Pain Medicine*, *Anaesthesiology Intensive Therapy*, *Anesthesia and Pain Medicine*, *Anasthesiologie und intensivmedizin*, *Anestezi Dergisi*, *Anesteziologie a Intenzivni Medicina*, *Anesthesiology*, *Annals of Critical Care*, *Best Practice & Research Clinical Anaesthesiology*, *Brazilian Journal of Anesthesiology*, *British Journal of Anaesthesia*, *Canadian Journal of Anesthesia-Journal Canadien d Anesthesie*, *Canadian Journal of Pain*, *Clinical Journal of Pain*, *Colombian Journal of Anesthesiology*, *Critical Care and Resuscitation*, *Current Opinion in Anesthesiology*, *Douleur et Analgesie*, *Indian Journal of Anaesthesia*, *Indian Journal of Clinical Anaesthesia*, *Indian Journal of Respiratory Care*, *International Journal of Obstetric Anesthesia*, *Journal of Anesthesia Analgesia and Critical Care*, *Journal of Cardiothoracic and Vascular Anesthesia*, *Journal of Clinical Anesthesia*, *Journal of Critical Care Medicine*, *Journal of Opioid Management*, *Journal of Pain*, *Journal of Pain Research*, *Journal of Palliative Medicine*, *Korean Journal of Anesthesiology*, *Minerva Anestesiologica*, *Open Pain Journal*, *Pain Research and Management*, *Palliative Medicine*, *Palliative Medicine Reports*, *Patient Safety in Surgery*, *Pediatric Anesthesia and Critical Care Journal*, *Perioperative Care and Operating Room Management*, *Regional Anesthesia and Pain Medicine*, *Revista Espanola de Anestesiologia y Reanimacion*, *Saudi Journal of Anaesthesia*, *Scandinavian Journal of Pain*, *Seminars in Arthritis and Rheumatism*, *Sri Lankan Journal of Anaesthesiology*, *The Journal of Headache and Pain*, *Trends in Anaesthesia and Critical Care*, *Turkish Journal of Anaesthesiology and Reanimation*, and *Update in Anaesthesia.*

Most of the workgroup’s journals publish exclusively in English, but some accept or publish in a bilingual format (including Czech, French, Portuguese, Russian, Slovak, Spanish, and Turkish). The workgroup is composed of members from all continents, with a predominance of North America (19 members) and Europe (18 members), followed by Asia (six members), Oceania (three members), South America (three members), and Africa (one member).

The first round included 44 members of the working group (83.0%), the second round 46 members (86.8%), and the third round 42 members (79.2%).Thirty-six members (67.9%) participated in all rounds. In contrast, three members (5.7%) did not participate in any round and were therefore not considered among the authors involved in drafting these recommendations.

### Statements

After the first round, a total of 61 statements were coded by the project administrators from the responses provided by 44 members of the working group. These statements were rated by the working group as accepted with strong consensus in 22 cases, accepted with consensus in 23 cases, 16 did not reach consensus, and none were rejected.

The 22 items accepted without strong consensus underwent minor rephrasing in Round 3, whereas the 16 items that did not reach consensus were revised according to the working group’s suggestions. In addition, three supplementary items were created in response to the panel’s feedback. In Round 3, 21 items reached strong consensus, 15 items reached consensus, and five items did not reach consensus. The optional fourth round was not conducted given that fewer than six items failed to reach consensus. The Delphi process yielded 59 statements, which have been organised into three categories for clarity: (1) general and author-related statements (Table 1), (2) editor, reviewer, and publisher-related statements (Table 2), and (3) statements concerning LLM disclosure and perceived risks (Table 3).

**Table 1:** General principles and author-focused statements. CONSORT, Consolidated Standards of Reporting Trials; GDPR, General Data Protection Regulation; HIPAA, Health Insurance Portability and Accountability Act; LLM, large language model; PRISMA, Preferred Reporting Items for Systematic reviews and Meta-Analyses; STROBE, Strengthening the Reporting of Observational Studies in Epidemiology.

**General** *Strong consensus* 1. This consensus should be treated as a living document, updated regularly to reflect emerging evidence, technology, and ethical developments. *Consensus* 2. Permitted, disclosed, and supervised use of LLMs, in line with each journal’s policy, does not diminish the scientific quality or integrity of a manuscript and should not be perceived negatively by authors, reviewers, or editors. **Authors** *Strong consensus* 3. LLMs cannot be listed as authors because they do not meet authorship criteria. 4. Human authors retain ultimate responsibility for the accuracy, integrity, and originality of manuscripts, even when LLMs are used for support. 5. All LLM contributions must be critically reviewed, verified, and endorsed by the authors before submission. 6. LLMs may be used for language editing—such as improving grammar, syntax, tone, and clarity. 7. The inclusion of any LLM-generated content is acceptable only when its creation and use are transparently disclosed and compliant with journal guidelines. 8. Formatting support, including reference styling, adherence to journal structure, and compliance with word limits, is an appropriate use of LLMs when transparently disclosed. 9. Abstracts, titles, or plain-language summaries may be drafted with LLM support, provided all content is reviewed and verified by the authors for accuracy and appropriateness. 10. Whenever LLMs are used to summarise a text, the outputs should always be verified against the original sources. 11. It is the author’s responsibility to ensure that figures or images created using generative tools are accurate and do not infringe copyright. 12. It is the author’s responsibility to ensure that layout or caption for figures and tables generated by LLMs are accurate. 13. LLMs may assist in refining search strategies (e.g. suggesting synonyms or Boolean operators) if their use is transparently disclosed. 14. LLMs must not be used to generate primary data or fabricate datasets. 15. Interpretation of results, drawing conclusions, and placing findings into context must remain human responsibilities. 16. LLMs should not be used to autonomously generate references or citations. 17. Patient-identifiable or confidential data must never be input into public or unsecured LLM platforms, as this poses significant ethical and legal risks under HIPAA, GDPR, and related regulations. 18. Entire manuscripts or sections drafted by LLMs are inappropriate, as this undermines intellectual authorship and may result in unverifiable content. 19. Hypothesis generation, especially for original research design, should remain a core intellectual responsibility of researchers. 20. Authors must ensure that any patient-specific recommendations generated by LLMs are accurate. 21. Authors must disclose all substantial uses of LLMs in manuscript preparation, specifying how and for what purposes the tools were applied. 22. Authors should not request LLMs to modify or rephrase copyrighted material without the explicit consent of the copyright holder. 23. The original source must always be properly cited, even when permission to use or adapt the material has been obtained. *Consensus* 24. LLMs may be used to support conceptual mapping as part of the brainstorming process. 25. Compliance checks with reporting standards (e.g. CONSORT, PRISMA, STROBE) are an appropriate supportive role for LLMs, provided such use is transparently disclosed, and supplemented by author review. 26. Manuscripts translated using automatic tools are acceptable only when their accuracy has been reviewed and confirmed by at least one author or by a professional translator fluent in the target language. 27. Before using LLMs to analyse patient data, authors must obtain approval from an institutional ethics committee to ensure patient privacy and compliance with applicable regulations.

**Table 2:** Editor-, reviewer-, and publisher-related statements. AI, artificial intelligence; LLM, large language model.

*Strong consensus* 1. Any prohibited use of LLMs should be treated as a serious breach, with grounds for manuscript rejection or retraction. 2. It is the responsibility of journals to clarify that LLMs must be acknowledged, with a description of their specific contribution, in the same way as professional language editing or translation services. 3. It is the responsibility of journals to establish clear instructions on the acceptable use of LLMs and on how they should be acknowledged. 4. Editorial decisions on novelty, impact, or acceptance of manuscripts based on LLM-generated content are not acceptable. 5. LLMs may assist editors in detecting plagiarism, duplicate submissions, or potential signs of AI-generated content. 6. LLMs may help flag internal inconsistencies (e.g. mismatches between abstracts, tables, and main text), but authors and editors must verify accuracy. 7. Screening for adherence to journal guidelines, including required sections, word counts, or formatting, can be appropriately automated or supported by LLMs. 8. Publishers and editors share responsibility for implementing transparent policies. 9. Peer review reports generated by LLMs are not acceptable, as they breach confidentiality. 10. Confidential manuscripts should only be processed with secure, journal-approved AI systems; uploading to public tools violates confidentiality obligations. 11. Editors and reviewers should disclose any use of LLMs in the assessment process, specifying which LLM was used and the acceptable tasks it supported. *Consensus* 12. LLMs should not be used as the sole method for identifying submissions with excessive AI-generated content. 13. Editorial workflows may include disclosed LLM-based tools for language polishing or reference formatting, provided their use is transparently disclosed. 14. LLMs may aid in identifying potential reviewers by screening databases for expertise and conflicts of interest however, editors must retain ultimate responsibility for reviewer assignment. 15. Summaries generated by LLMs may assist reviewers in triaging manuscripts, provided the LLM is approved for this task by the journal, and final evaluations remain fully dependent on human review. 16. Reviewers may use LLMs to improve the clarity or language of their feedback; however, they remain fully responsible for the content and integrity of their report.

**Table 3:** LLM disclosure and perceived risks. AI, artificial intelligence; LLM, large language model.

**LLM disclosure** *Strong consensus* 1. Failure to transparently disclose the use of LLMs in manuscript preparation undermines trust, and deliberate deception regarding such use constitutes academic misconduct. 2. A standardised ‘AI Disclosure Statement’ section, similar to conflict-of-interest declarations, should be added to manuscripts to harmonise reporting. 3. Any use of LLMs related to the research methodology should be transparently reported in the Methods section. 4. Disclosure statements should specify the tool name, version, access date, and scope of use. 5. Journals should provide authors with standardised disclosure templates to ensure uniformity across submissions. 6. Publishers and journals should emphasise that disclosure promotes transparency, trust, and responsible use—not punishment. *Consensus* 7. Authors are encouraged to disclose the use of LLMs in the cover letter, indicating the nature, extent, and purpose of their contribution. 8. Single words or short sentences spelling or punctuation checks do not require disclosure, similar to conventional grammar software. 9. Authors are encouraged to document LLM prompts, parameters, and workflows for transparency and accountability. **Perceived risks** *Strong consensus* 1. LLMs are prone to generating ‘hallucinations’, such as fabricated data, references, or plausible but false statements, which could mislead readers. 2. Over-reliance on LLMs without adequate training may erode essential scholarly skills, including critical thinking, originality, and scientific writing. 3. LLM use may inadvertently expose confidential or unpublished material, risking intellectual property breaches. 4. The proliferation of low-quality AI-generated manuscripts risks overwhelming peer review and diminishing publication standards. 5. Training in the use of LLMs should be considered part of responsible academic practice. *Consensus* 6. Outputs from LLMs are not predictable; the same prompt can yield different responses depending on the model version or context, complicating reproducibility. 7. Uncontrolled growth of AI-generated manuscripts could make future AI tools train on their own artificial content, which may cause ‘model collapse’, lowering the quality of scientific output.

## Discussion

### General principles and author-focused statements

The working group emphasises that it is expected that journals will provide clear guidance on acceptable LLM use, recognising that their appropriate role lies in assisting—but not replacing—human scholarly activity. All parties are obligated to safeguard confidential materials, ensure accountability, and disclose any use of LLM tools.

Responsible and transparent use of LLMs can support scientific writing without lowering manuscript quality or compromising research integrity, provided these tools are used for supportive or derivative tasks that do not require original intellectual contribution.

Human authors should retain full accountability for the accuracy, originality, and interpretation of all content. Appropriate uses of LLMs include language editing, formatting, summarisation, translation, search-strategy refinement, and compliance checks—provided all outputs are carefully verified and fully disclosed.

By contrast, the working group draws a clear boundary at tasks that involve original scholarly judgement or intellectual ownership. LLMs should not be used for ideative or conceptual contributions that define the novelty, argument, or scientific direction of a work, as these responsibilities must remain strictly human. LLMs must not be used to generate primary data, references, conclusions, or—most critically—entire manuscripts. Particular care is required to protect confidential data and avoid misuse of copyrighted material. Responsibility for the appropriate and compliant use of LLM tools is distributed across the scholarly publishing process: authors are required to exercise due diligence in their use of these tools, whereas publishers and editorial offices play an important role in providing clear guidance and oversight to support copyright compliance at the publication stage.

In the context of *in silico* or computational modelling, it might be tempting to use LLMs to generate synthetic datasets. However, unlike statistical software that produces controlled and reproducible datasets, current LLMs do not provide guarantees regarding true randomness, reproducibility, or the characteristics of the generated data. Consequently, their use for producing primary datasets, even in this context, is strongly discouraged by the working group.

Some applications—such as conceptual mapping, assistance with reporting guideline compliance, and certain forms of summarisation—reached consensus but not strong consensus probably because these uses occupy a methodological ‘grey zone’ between technical assistance and intellectual contribution. Therefore, authors are called to exercise particular caution when using LLMs for these purposes, carefully reviewing journal policies to ensure that their practices remain aligned with current knowledge and expectations.

### Editor, reviewer, and publisher statements

Editors, peer reviewers, and publishers share responsibility for ensuring the transparent, ethical, and appropriate use of LLMs throughout the editorial process.

The working group emphasised that responsible and transparent use of LLMs can enhance the editorial process without compromising manuscript quality or research integrity, as long as human judgement remains central. In particular, LLMs may support tasks that are derivative or administrative in nature, such as language polishing, reference formatting, plagiarism checks, or identifying inconsistencies, with their use fully disclosed to maintain transparency. They can also assist editors in screening potential reviewers for expertise and conflicts of interest, or help reviewers triage manuscripts by summarising content, but ultimate decisions and evaluations must always remain with humans. Similarly, reviewers may use LLMs to refine the language and improve the clarity of their feedback, yet they retain full responsibility for the accuracy, appropriateness, and integrity of their reports.

However, the group agreed that reliance on LLMs must be prohibited for core editorial decisions, generating peer-review reports, or any task that may compromise confidentiality or scientific integrity.

Importantly, some statements—particularly those concerning the use of LLMs for manuscript triage and for improving the language of reviewers’ reports—reached consensus but not strong consensus. These areas similarly represent a boundary zone where assistance may be helpful but risks over-automation. For this reason, the working group urges particular caution when using LLMs for these purposes and recommends that any such use in editorial or peer-review workflows be fully disclosed and strictly aligned with each journal’s policies.

### Large language model use disclosures

The statements endorsed by the working group emphasise that transparent disclosure of LLM use should be encouraged, because it promotes trust in the authors and does not diminish the quality of the work. Deliberate nondisclosure, by contrast, may constitute academic misconduct.

A standardised ‘Artificial intelligence (AI) disclosure section’ should be included in manuscripts. This should clearly report the tool name and version, access date, and the specific role of the LLM in manuscript preparation or research methodology. Authors are encouraged to disclose LLM use as early as in the cover letter and to document prompts to enhance reproducibility, although these practices did not receive strong consensus across the entire working group. The group also considers it valuable for journals to adopt uniform disclosure templates.

### Perceived risks

The working group highlights several potential risks associated with LLM use; however, provided that this working group is composed solely of EIC and their delegates—not experts in AI or ethics—their perception on potential risks should be interpreted with caution and primarily as the viewpoint of editors who encounter this technology in their daily editorial workflow.

It is the opinion of the working group that contemporary LLMs can produce ‘hallucinations’, such as fabricated data or references, and that their outputs are unpredictable, which, combined with over-reliance on these tools, may erode critical scholarly skills and mislead authors and readers. Use of LLMs also carries risks for confidential or unpublished material, whereas the growth of low-quality AI-generated manuscripts could overwhelm peer review and feed future models, potentially leading to ‘model collapse’. For these reasons, education in the responsible use of LLMs is of paramount importance.

### Comparison of RULE-AP with other published policies on large language model use

RULE-AP is not the first attempt to produce clear guidelines on the use of LLMs in academic publishing. The statements from RULE-AP align with the Committee on Publication Ethics (COPE) in noting that LLM tools cannot be listed as authors on scientific papers.^14^ Moreover, a recent Regional Anesthesia & Pain Medicine (RAPM) consensus was developed by the RAPM editorial board, offering valuable recommendations on disclosure, human accountability, and the use of AI-detection tools. However, its scope is broader, addressing AI in general rather than focusing specifically on LLMs. It incorporated a broader set of experts in AI. The unique contribution of RULE-AP is the focus specifically on the viewpoints of EIC in anaesthesia and pain medicine and related fields with assertions that apply only to the use of LLMs.^15^

In a broader context, an *Editors’ Statement on the Responsible Use of Generative AI* endorsed by several editors in bioethics^16^ warns against relying solely on AI for peer review or editorial decisions and calls for transparency about how AI is used and disclosed. However, unlike RULE-AP, it was not developed using a structured consensus method such as a Delphi process, resulting in guidance that is principled but not methodologically standardised.

The CANGARU guideline project (ChatGPT, Generative AI, and Natural LLMs for Accountable Reporting and Use) aims to create cross-disciplinary, standardised reporting norms for AI-assisted research, stressing documentation, accountability, and continuous updating of standards.^17^ However, CANGARU remains a proposed, ongoing project rather than a released, validated guideline, and therefore currently provides conceptual direction rather than a specific operational guideline.

### Study limitations

This Delphi consensus reflects the perspectives of EICs and their delegates from anaesthesiology and pain medicine journals and does not include experts in AI, ethics, or broader scientific publishing. Consequently, the identified risks and recommended practices should be interpreted as the viewpoint of journal editors managing LLMs in their daily editorial workflows, rather than as definitive, evidence-based recommendations for the wider community of authors, reviewers, and publishers. The participation rate (36.0% of eligible journals) may introduce selection bias, and although the Delphi method facilitates structured consensus, it remains inherently subjective and dependent on the expertise and experiences of participants. Finally, the rapidly evolving nature of LLM technology means that guidance will likely need regular updating to remain relevant and aligned with emerging evidence and ethical standards.

### Conclusions

The RULE-AP Delphi project provides editor-informed guidance for the responsible use of LLMs in manuscript preparation, peer review, and editorial workflows. Key principles include transparency, human accountability, and human verification of all tasks and outputs. This guidance reflects current knowledge but may quickly be superseded as technology and understanding evolve. Additional caution is recommended for areas lacking strong consensus.

## Authors’ contributions

Data curation, investigation, project administration: ADC

Conceptualisation, methodology, supervision, writing—original draft: ADC, BD

Investigation, validation: all other members of the Delphi working group

Writing—review and editing: all authors

## Declarations of interest

HCH is the editor-in-chief of the *British Journal of Anaesthesia*. He had no involvement in, and exerted no influence over, the editorial handling or peer-review process of this manuscript. All panellists serve as editors-in-chief of journals classified in ‘anaesthesia’ or ‘pain medicine’ categories by Scopus or Web of Science or have been formally designated by their respective editors-in-chief to represent their journals on this panel. MKF is the editor-in-chief of *Best Practice & Research Clinical Anaesthesia* for which she receives a stipend from Elsevier. She serves as an advisory board member for Octapharma and receives royalties from Wolters Kluwer as an author for UpToDate. She receives research funding from Flat Medical and Aerobiotix. AAG has received compensation from MSD, 3M-Solventum, and Altan-Ethipharm for conference-related activities. MCH, as editor-in-chief of *Seminars in Arthritis and Rheumatism*, receives a stipend from Elsevier, Inc. In addition, he receives royalties from Wolters Kluwer as a reviewer for UpToDate. SKWS is the editor-in-chief of the *Canadian Journal of Anesthesia/Journal Canadien d’Anesthésie* for which he receives consultant fees from the Canadian Anesthesiologists’ Society, and holds the Dr Jean Templeton Hugill Chair in Anesthesia, supported by the Dr Jean Templeton Hugill Endowment of Anesthesia Memorial Fund (Faculty of Medicine, The University of British Columbia, Vancouver, BC, Canada); he has no financial relationships with commercial entities. TMP is editor-in-chief of *The Journal of Pain* for which she receives a stipend from the US Association for the Study of Pain, receives research grant funding from the National Institutes of Health, receives book royalties from Oxford University Press, and provides consultation to TriveniBio. PJP is the editor-in-chief of the *Anaesthesia and Intensive Care* for which he receives remuneration from the Australian Society of Anaesthetists; he has no current financial relationships with commercial entities. BS is the Executive Editor of A & A Practice and receives funding from IARS and royalties from Wolters Kluwer as an author for UpToDate. DCT, editor-in-chief of the *Clinical Journal of Pain*, is Associate Director of Analgesic, Anesthetic, and Addiction Clinical Trial Translations, Innovations, Opportunities, and Networks (ACTTION) but has no financial relationships with any commercial entities. The other authors declare that they have no conflicts of interest. PM serves as Editor-in-Chief of The Journal of Headache and Pain and receives annual financial support from the publisher, Springer Nature.
