# Supplementary material for: Responsible use of large language models in manuscript authorship, peer review, and editorial processes: a Delphi consensus among editors-in-chief of anaesthesia and pain medicine journals (RULE-AP)
Source: Br J Anaesth. 2026 Feb 26;136(5):1625–33. doi: 10.1016/j.bja.2026.01.029 (PMC13639462; doi:10.1016/j.bja.2026.01.029)
Supplement: Supplementary file 1 [file mmc1.docx]

**Responsible Use of Large language models in Editorial practice: a delphi consensus among editors-in-chief of Anesthesiology and Pain medicine journals (RULE-AP)**

–Supplementary material–

Table of contents

Identified eligible journals	page 2

Participating Editors-in-Chief or their delegates	page 9

Round 1 – Open-Ended Questionnaire	page 11

Round 2 – Statements and Voting Results	page 13

Round 3 – Statements and Voting Results	page 19 Items evaluation over individual rounds	page 24

CREDES checklist	page 27

**Identified eligible journals**

| Number | Journal (alphabetical order) |
| --- | --- |
| 1 | A & A PRACTICE |
| 2 | AANA JOURNAL |
| 3 | ACTA ANAESTHESIOLOGICA BELGICA |
| 4 | ACTA ANAESTHESIOLOGICA SCANDINAVICA |
| 5 | ACTA COLOMBIANA DE CUIDADO INTENSIVO |
| 6 | ACUPUNCTURE AND ELECTRO-THERAPEUTICS RESEARCH |
| 7 | ADVANCES IN ANESTHESIA |
| 8 | AGRI |
| 9 | AIN SHAMS JOURNAL OF ANESTHESIOLOGY |
| 10 | AMBULATORNAYA KHIRURGIYA |
| 11 | ANAESTHESIA |
| 12 | ANAESTHESIA AND INTENSIVE CARE |
| 13 | ANAESTHESIA AND INTENSIVE CARE MEDICINE |
| 14 | ANAESTHESIA CRITICAL CARE & PAIN MEDICINE |
| 15 | ANAESTHESIA PAIN & INTENSIVE CARE |
| 16 | ANAESTHESIA REPORTS |
| 17 | ANAESTHESIA, PAIN AND INTENSIVE CARE |
| 18 | ANAESTHESIOLOGIE |
| 19 | ANAESTHESIOLOGY INTENSIVE THERAPY |
| 20 | ANASTHESIOLOGIE INTENSIVMEDIZIN NOTFALLMEDIZIN SCHMERZTHERAPIE |
| 21 | ANASTHESIOLOGIE UND INTENSIVMEDIZIN |
| 22 | ANESTEZI DERGISI |
| 23 | ANESTEZIOLOGIE A INTENZIVNI MEDICINA |
| 24 | ANESTHESIA AND ANALGESIA |
| 25 | ANESTHESIA AND PAIN MEDICINE |
| 26 | ANESTHESIA PROGRESS |
| 27 | ANESTHESIE & REANIMATION |
| 28 | ANESTHESIOLOGY |
| 29 | ANESTHESIOLOGY AND PAIN MEDICINE |
| 30 | ANESTHESIOLOGY CLINICS |
| 31 | ANESTHESIOLOGY RESEARCH AND PRACTICE |
| 32 | ANNALS OF CARDIAC ANAESTHESIA |
| 33 | ANNALS OF CRITICAL CARE |
| 34 | ARCHIVES OF ANESTHESIOLOGY AND CRITICAL CARE |
| 35 | ASIAN JOURNAL OF ANESTHESIOLOGY |
| 36 | BALI JOURNAL OF ANESTHESIOLOGY |
| 37 | BEST PRACTICE & RESEARCH-CLINICAL ANAESTHESIOLOGY |
| 38 | BJA EDUCATION |
| 39 | BJA OPEN |
| 40 | BMC ANESTHESIOLOGY |
| 41 | BRAZILIAN JOURNAL OF ANESTHESIOLOGY |
| 42 | BRITISH JOURNAL OF ANAESTHESIA |
| 43 | BRITISH JOURNAL OF PAIN |
| 44 | CANADIAN JOURNAL OF ANESTHESIA-JOURNAL CANADIEN D’ANESTHESIE |
| 45 | CANADIAN JOURNAL OF PAIN |
| 46 | CASE REPORTS IN ANESTHESIOLOGY |
| 47 | CHINESE JOURNAL OF ANESTHESIOLOGY |
| 48 | CLINICAL JOURNAL OF PAIN |
| 49 | COLOMBIAN JOURNAL OF ANESTHESIOLOGY |
| 50 | CRITICAL CARE AND RESUSCITATION |
| 51 | CURRENT ANESTHESIOLOGY REPORTS |
| 52 | CURRENT OPINION IN ANESTHESIOLOGY |
| 53 | CURRENT PAIN AND HEADACHE REPORTS |
| 54 | DOLOR |
| 55 | DOULEUR ET ANALGESIE |
| 56 | DOULEURS |
| 57 | EGYPTIAN JOURNAL OF ANAESTHESIA |
| 58 | EMERGENCY MEDICINE (UKRAINE) |
| 59 | EUROPEAN JOURNAL OF ANAESTHESIOLOGY |
| 60 | EUROPEAN JOURNAL OF ANAESTHESIOLOGY AND INTENSIVE CARE |
| 61 | EUROPEAN JOURNAL OF PAIN |
| 62 | FRONTIERS IN PAIN RESEARCH |
| 63 | HIRURGIA POZVONOCHNIKA |
| 64 | INDIAN ANAESTHETISTS FORUM |
| 65 | INDIAN JOURNAL OF ANAESTHESIA |
| 66 | INDIAN JOURNAL OF CLINICAL ANAESTHESIA |
| 67 | INDIAN JOURNAL OF RESPIRATORY CARE |
| 68 | INTERNATIONAL ANESTHESIOLOGY CLINICS |
| 69 | INTERNATIONAL JOURNAL OF OBSTETRIC ANESTHESIA |
| 70 | INTERVENTIONAL PAIN MEDICINE |
| 71 | JA CLINICAL REPORTS |
| 72 | JAMS JOURNAL OF ACUPUNCTURE AND MERIDIAN STUDIES |
| 73 | JOURNAL OF ANAESTHESIOLOGY CLINICAL PHARMACOLOGY |
| 74 | JOURNAL OF ANESTHESIA |
| 75 | JOURNAL OF ANESTHESIA ANALGESIA AND CRITICAL CARE |
| 76 | JOURNAL OF ANESTHESIA AND TRANSLATIONAL MEDICINE |
| 77 | JOURNAL OF CARDIOTHORACIC AND VASCULAR ANESTHESIA |
| 78 | JOURNAL OF CELLULAR AND MOLECULAR ANESTHESIA |
| 79 | JOURNAL OF CLINICAL ANESTHESIA |
| 80 | JOURNAL OF CLINICAL MONITORING AND COMPUTING |
| 81 | JOURNAL OF CRITICAL CARE MEDICINE |
| 82 | JOURNAL OF DENTAL ANESTHESIA AND PAIN MEDICINE |
| 83 | THE JOURNAL OF HEADACHE AND PAIN |
| 84 | JOURNAL OF NEUROANAESTHESIOLOGY AND CRITICAL CARE |
| 85 | JOURNAL OF NEUROSURGICAL ANESTHESIOLOGY |
| 86 | JOURNAL OF OBSTETRIC ANAESTHESIA AND CRITICAL CARE |
| 87 | JOURNAL OF OPIOID MANAGEMENT |
| 88 | JOURNAL OF ORAL AND FACIAL PAIN AND HEADACHE |
| 89 | JOURNAL OF PAIN |
| 90 | JOURNAL OF PAIN & PALLIATIVE CARE PHARMACOTHERAPY |
| 91 | JOURNAL OF PAIN AND SYMPTOM MANAGEMENT |
| 92 | JOURNAL OF PAIN RESEARCH |
| 93 | JOURNAL OF PALLIATIVE MEDICINE |
| 94 | JOURNAL OF PERIOPERATIVE PRACTICE |
| 95 | JOURNAL OF RESEARCH IN CLINICAL MEDICINE |
| 96 | KOREAN JOURNAL OF ANESTHESIOLOGY |
| 97 | KOREAN JOURNAL OF PAIN |
| 98 | LOCAL AND REGIONAL ANESTHESIA |
| 99 | MEDECINE PALLIATIVE |
| 100 | MEDICAL GAS RESEARCH |
| 101 | MEDICINA PALIATIVA |
| 102 | MESSENGER OF ANESTHESIOLOGY AND RESUSCITATION |
| 103 | MIDDLE EAST JOURNAL OF ANESTHESIOLOGY |
| 104 | MINERVA ANESTESIOLOGICA |
| 105 | MOLECULAR PAIN |
| 106 | NEUROBIOLOGY OF PAIN |
| 107 | NEUROMODULATION |
| 108 | OPEN ANESTHESIA JOURNAL |
| 109 | OPEN PAIN JOURNAL |
| 110 | PAIN |
| 111 | PAIN AND THERAPY |
| 112 | PAIN MANAGEMENT |
| 113 | PAIN MEDICINE |
| 114 | PAIN PHYSICIAN |
| 115 | PAIN PRACTICE |
| 116 | PAIN REPORTS |
| 117 | PAIN RESEARCH AND MANAGEMENT |
| 118 | PAIN, JOINTS, SPINE |
| 119 | PALLIATIVE MEDICINE |
| 120 | PALLIATIVE MEDICINE REPORTS |
| 121 | PATIENT SAFETY IN SURGERY |
| 122 | PEDIATRIC ANESTHESIA |
| 123 | PEDIATRIC ANESTHESIA AND CRITICAL CARE JOURNAL |
| 124 | PERIOPERATIVE CARE AND OPERATING ROOM MANAGEMENT |
| 125 | PERIOPERATIVE MEDICINE |
| 126 | PRATICIEN EN ANESTHESIE REANIMATION |
| 127 | PRECISION CANCER MEDICINE |
| 128 | REGIONAL ANESTHESIA AND ACUTE PAIN MANAGEMENT |
| 129 | REGIONAL ANESTHESIA AND PAIN MEDICINE |
| 130 | REVISTA CHILENA DE ANESTESIA |
| 131 | REVISTA DE LA SOCIEDAD ESPANOLA DEL DOLOR |
| 132 | REVISTA ESPANOLA DE ANESTESIOLOGIA Y REANIMACION |
| 133 | REVISTA MEXICANA DE ANESTESIOLOGIA |
| 134 | RUSSIAN JOURNAL OF ANESTHESIOLOGY AND REANIMATOLOGY /ANESTEZIOLOGIYA I REANIMATOLOGIYA |
| 135 | RUSSIAN JOURNAL OF PEDIATRIC SURGERY, ANESTHESIA AND INTENSIVE CARE |
| 136 | SAUDI JOURNAL OF ANAESTHESIA |
| 137 | SCANDINAVIAN JOURNAL OF PAIN |
| 138 | SCHMERZ |
| 139 | SEMINARS IN ARTHRITIS AND RHEUMATISM |
| 140 | SEMINARS IN CARDIOTHORACIC AND VASCULAR ANESTHESIA |
| 141 | SOUTHERN AFRICAN JOURNAL OF ANAESTHESIA AND ANALGESIA |
| 142 | SRI LANKAN JOURNAL OF ANAESTHESIOLOGY |
| 143 | THERAPEUTIC HYPOTHERMIA AND TEMPERATURE MANAGEMENT |
| 144 | TRENDS IN ANAESTHESIA AND CRITICAL CARE |
| 145 | TURKISH JOURNAL OF ANAESTHESIOLOGY AND REANIMATION |
| 146 | ULUSAL TRAVMA VE ACIL CERRAHI DERGISI |
| 147 | UPDATE IN ANAESTHESIA |

**Participating Editors-in-Chief or Delegates (Listed Alphabetically by Journal)**

1. BobbieJean Sweitzer, EIC of *A & A Practice*
2. Stefan De Hert, EIC of *Acta Anaesthesiologica Belgica*
3. Gül Köknel Talu, EIC of *Agri*
4. Philip J. Peyton, EIC of *Anaesthesia and Intensive Care*
5. Antoine Roquilly, EIC of *Anaesthesia Critical Care & Pain Medicine*
6. Szymon Zdanowski delegated by EIC of *Anaesthesiology Intensive Therapy*
7. Hyun Kang, EIC of Anesthesia and *Pain Medicine*
8. Zerrin Özköse Şatırlar, EIC of *Anestezi Dergisi*
9. Vladimír Černý delegated by Jiří Málek (EIC) of *Anesteziologie a Intenzivni Medicina*
10. James P. Rathmell, EIC of *Anesthesiology*
11. Igor Borisovich Zabolotskikh, EIC of *Annals of Critical Care*
12. Michaela K. Farber, EIC of *Best Practice & Research-Clinical Anaesthesiology*
13. Hugh C. Jr Hemmings, EIC of *British Journal of Anaesthesia*
14. Liana Maria Torres de Araújo Azi, EIC of *Brazilian Journal of Anesthesiology*
15. Stephan K. W. Schwarz, EIC of *Canadian Journal of Anesthesia-Journal canadien d anesthesie*
16. Joel D. Katz, EIC of *Canadian Journal of Pain*
17. Dennis C. Turk, EIC of *Clinical Journal of Pain*
18. José Andrés Calvache, EIC of *Colombian Journal of Anesthesiology*
19. Ary Serpa Neto, EIC of *Critical Care and Resuscitation*
20. Paul J. Young, EIC of *Critical Care and Resuscitation*
21. Kai Zacharowski, EIC of *Current Opinion in Anesthesiology* and *Anasthesiologie und intensivmedizin*
22. Pierrick Poisbeau, EIC of *Douleur et Analgesie*
23. Rakesh Garg, EIC of Indian *Journal of Anaesthesia*
24. Lalit Gupta, EIC of Indian *Journal of Clinical Anaesthesia*
25. Madhuragauri Shevade, EIC of *Indian Journal of Respiratory Care*
26. Ruth Landau, EIC of *International Journal of Obstetric Anesthesia*
27. John G. Augoustides, delegated by Joel A. Kaplan, EIC of *Journal of Cardiothoracic and Vascular Anesthesia*
28. Alessandro De Cassai, delegated by Giorgio Conti, EIC of *Journal of Anesthesia Analgesia and Critical Care*
29. Alparslan Turan, EIC of *Journal of Clinical Anesthesia*
30. Leonard Azamfirei, EIC o*f Journal of Critical Care Medicine*
31. Paolo Martelletti, EIC of *The Journal of Headache and Pain*
32. Paul A. Sloan, EIC of *Journal of Opioid Management*
33. Tonya M. Palermo, EIC o*f Journal of Pain*
34. Michael Schatman, EIC of *Journal of Pain Research*
35. Christopher A. Jones, EIC of Journal of Palliative Medicine
36. Sangseok Lee, EIC of *Korean Journal of Anesthesiology*
37. Marco Rossi, delegated by Franco Cavaliere, EIC of *Minerva Anestesiologica*
38. Durval C. Kraychete, EIC of *Open Pain Journal*
39. Amara Nwosu, delegated by Catherine Walshe, EIC of *Palliative Medicine*
40. Hillary Lum, EIC of *Palliative Medicine Reports*
41. Massimiliano Valeriani, EIC of *Pain Research and Management*
42. Philip F. Stahel, EIC of *Patient Safety in Surgery*
43. Dario Galante, EIC of *Pediatric Anesthesia and Critical Care Journal*
44. Heidi V. Goldstein delegated by Mitchell Tsai, EIC of *Perioperative Care and Operating Room Management*
45. Michael R Fettiplace delegated by Brian D. Sites, EIC of *Regional Anesthesia and Pain Medicine*
46. Alfredo Abad-Gurumeta, EIC of *Revista Espanola de Anestesiologia y Reanimacion*
47. Abdelazeem Eldawlatly, EIC of *Saudi Journal of Anaesthesia*
48. Mads U. Werner, EIC of *Scandinavian Journal of Pain*
49. Marc C. Hochberg, EIC of *Seminars in Arthritis and Rheumatism*
50. Thamasi Rekha Makuloluwa, EIC of *Sri Lankan Journal of Anaesthesiology*
51. Diogo Sobreira-Fernandes, EIC of *Trends in Anaesthesia and Critical Care*
52. Zekeriyya Alanoğlu, EIC of *Turkish Journal of Anaesthesiology and Reanimation*
53. Christina Lundgren, EIC of U*pdate in Anaesthesia*

# **Round 1 – Open-Ended Questionnaire**

**Introduction for participants:**
 You are invited to participate in the first round of a Delphi study exploring the use of large language models (LLMs), such as ChatGPT or Claude, in academic manuscript preparation. The purpose of this round is to gather your perspectives on multiple domains of relevance, including ethical considerations, authorship, acceptable uses, disclosure practices, and editorial workflows.

There are no right or wrong answers. Please provide as much detail as you consider relevant. Your responses will be anonymized and used to generate structured items for subsequent rounds. Completion time: approximately 15–20 minutes.

## **Section A — General Perspectives**

1. In your view, what are the main opportunities and benefits of using LLMs in academic manuscript preparation in anesthesiology and pain medicine?
2. What are the main risks, concerns, or potential harms associated with their use?

## **Section B — Ethics and Authorship**

3. How should the use of LLMs be considered with respect to authorship? Should they be acknowledged, credited as authors, or neither? Please explain your reasoning.
4. What ethical principles should guide the use of LLMs in manuscript preparation?

## **Section C — Acceptable Use**

5. In which specific stages of manuscript preparation do you think LLMs could be used appropriately? Consider examples such as: idea generation, literature searching, drafting text, language editing, formatting, figure or table assistance, or summarizing content for systematic reviews.
6. In which stages or tasks do you think LLM use should be restricted or prohibited, and why?
7. Are there tasks in which LLMs could enhance peer review or editorial processes? If so, which ones, and under what conditions?

## **Section D — Disclosure and Transparency**

8. How should authors disclose the use of LLMs in their manuscripts, if at all? Consider options such as cover letters, acknowledgements, or methods sections.
9. Should journals establish standardized policies for LLM disclosure? If yes, what elements would be most important to include?

## **Section E — Additional Considerations**

10. Are there other important considerations regarding accuracy, bias, reproducibility, patient privacy, or data protection when using LLMs in manuscript preparation?
11. What practical guidance or editorial support would you like journals to provide authors regarding LLM use?
12. Are there any emerging issues, risks, or opportunities related to LLMs in academic publishing that you think should be included in a consensus statement?

## **Section F — Optional Contextual Information**

13. Your editorial role (Editor-in-Chief, Associate/Section Editor, other)
14. Years of editorial experience
15. Name of journal (optional, will be anonymized in analysis)

**Round 2 – Statements and Voting Results**

SC: Strong consensus; C: Consensus; NC: No Consensus; CA: Consensus against

Item 1: Large language models (LLMs) cannot be listed as authors because they do not meet authorship criteria.
**(SC)** **Median 9 (8-9) IQR 1; voted 46, >≥7 44(95.7%)**

Item 2: Human authors retain ultimate responsibility for the accuracy, integrity, and originality of manuscripts, even when LLMs are used for support.
**(SC) Median 9 (9 -9 ) IQR 0; voted 45; ≥7:45(100%)**

Item 3: All LLM contributions must be critically reviewed, verified, and endorsed by the authors before submission.
**(SC) Median 9 (9 -9 ) IQR 0 ; voted 46 ; ≥7:46(100%)**

Item 4: Undisclosed or deceptive use of LLMs in manuscript preparation undermines trust and constitutes a form of academic misconduct.

**(C) Median 9 (7 -9 ) IQR 2 ; voted 46 ; ≥7: 39(84.8%)**

Item 5: It is the responsibility of journals to clarify that LLMs may be acknowledged in the same way as professional language editing or translation services.
**(C) Median 8(7-9 ) IQR 2 ; voted 46 ; ≥7: 38( 82.6%)**

Item 6: It is the responsibility of journals to establish clear instructions on the acceptable use of LLMs and on how they should be acknowledged.
**(SC) Median 9(8 -9 ) IQR 1 ; voted 46 ; ≥7: 44(95.7%)**

Item 7: Outputs generated by LLMs can be used only if transparently disclosed.
**(C) Median 8.5 (7 -9 ) IQR 2 ; voted 46 ; ≥7: 35(76.1%)**

Item 8: The use of LLMs for permitted purposes does not diminish the scientific quality or integrity of a manuscript and should not be perceived negatively by authors, peer reviewers, or editors.
**(NC) Median 7(6 -9 ) IQR 3 ; voted 46 ; ≥7: 32(69.6%)**

Item 9: LLMs may be used for language editing—such as improving grammar, syntax, tone, and clarity.
**(SC) Median 9(8 -9 ) IQR 1 ; voted 46 ; ≥7:45(97.8%)**

Item 10: Formatting support, including reference styling, adherence to journal structure, and compliance with word limits, is an appropriate use of LLMs.
**(C) Median 8.5 (7 -9 ) IQR 2 ; voted 46 ; ≥7:41(89.1%)**

Item 11: Summarizing large volumes of text (e.g., background literature or extracted data in systematic reviews) is appropriate if outputs are verified against primary sources.
**(NC) Median 7(6 -8 ) IQR 2 ; voted 46 ; ≥7: 30(65.2%)**

Item 12: LLMs may be used to suggest layouts or captions for figures and tables.
**(NC) Median 8 (6 -9 ) IQR 3 ; voted 46 ; ≥7: 32(69.6%)**

Item 13: Brainstorming and idea generation are acceptable uses of LLMs.
**(NC) Median 7(4.25 -8 ) IQR 3.75 ; voted 46; ≥7: 26(56.5%)**

Item 14: LLMs may assist in refining search strategies (e.g., suggesting synonyms or Boolean operators).
**(C) Median 8 (7 -9 ) IQR 2 ; voted 45 ; ≥7: 39(86.7%)**

Item 15: Abstracts, titles, or plain-language summaries may be drafted with LLM support.
**(NC) Median 7 (5.25 -8.75 ) IQR 3.5 ; voted 46 ; ≥7: 31(67.4%)**

Item 16: Compliance checks with reporting standards (e.g., CONSORT, PRISMA, STROBE) are an appropriate supportive role for LLMs.
**(C) Median 7(7 -9 ) IQR 2 ; voted 46 ; ≥7:35(76.1%)**

Item 17: Automatic translation of manuscripts into other languages is acceptable only if at least one author, or a professional translator engaged by the authors, is proficient in the translated language.
**(C) Median 7(7 -9 ) IQR 2 ; voted 46 ; ≥7: 36(78.3%)**

Item 18: Journals should consider providing institution-approved or private LLM tools to allow authors to use them safely without risking confidentiality and to reduce disparities in access.
**(NC) Median 7(6 -9 ) IQR 3 ; voted 46 ; ≥7: 33(71.7%)**

Item 19: LLMs must not be used to generate or manipulate primary data, conduct statistical analyses, or fabricate datasets or case reports.
**(C) Median 9 (7.25 - 9 ) IQR 1.75 ; voted 46 ; ≥7: 38(82.6%)**

Item 20: Interpretation of results, drawing conclusions, and placing findings into context must remain human responsibilities.
**(SC) Median 9 (8 -9 ) IQR 1 ; voted 46 ; ≥7: 41(89.1%)**

Item 21: LLMs should not be used to autonomously generate references or citations.
**(SC) Median 9(8 -9 ) IQR 1 ; voted 46 ; ≥7: 39(84.8%)**

Item 22: Patient-identifiable or confidential data must never be input into public or unsecured LLM platforms, as this poses significant ethical and legal risks under HIPAA, GDPR, and related regulations.
**(SC) Median 9 (9 -9 ) IQR 0 ; voted 45 ; ≥7:45(100%)**

Item 23: Entire manuscripts or sections drafted by LLMs are inappropriate, as this undermines intellectual authorship and may result in unverifiable content.
**(SC) Median 9 (8 -9 ) IQR 1 ; voted 45 ; ≥7: 39(86.7%)**

Item 24: Hypothesis generation, especially for original research design, should remain a core intellectual responsibility of researchers.
**(SC) Median 9 (8 -9 ) IQR 1 ; voted 45 ; ≥7: 39(86.7%)**

Item 25: Figures or images created by generative tools may risk copyright infringement or misrepresentation.
**(C) Median 8 (7 -9 ) IQR 2 ; voted 45 ; ≥7:34(75.6%)**

Item 26: Using LLMs for clinical recommendations or patient-specific guidance is inappropriate and potentially harmful.
**(C) Median 8(6.25 -9 ) IQR 2.75 ; voted 46 ; ≥7:34(73.9%)**

Item 27: Peer review reports generated by LLMs are not acceptable, as they breach confidentiality and lack scholarly expertise.
**(C) Median 8(7 -9 ) IQR 2 ; voted 45 ; ≥7:37(82.2%)**

Item 28: Editorial decisions on novelty, impact, or acceptance of manuscripts based on LLM-generated content are not acceptable.
**(SC) Median 9 (8 -9 ) IQR 1 ; voted 44; ≥7: 37(84.1%)**

Item 29: LLMs may assist editors in detecting plagiarism, duplicate submissions, or potential signs of AI-generated content.
**(SC) Median 9(8 -9 ) IQR 1 ; voted 44 ; ≥7:40(90.0%)**

Item 30: LLMs should not be relied upon to detect submissions with excessive AI-generated content, as this approach is currently unreliable.
**(NC) Median 7 (6 -9 ) IQR 3 ; voted 44 ; ≥7:24(54.5%)**

Item 31: Screening for adherence to journal guidelines, including required sections, word counts, or formatting, can be appropriately automated or supported by LLMs.
**(SC) Median 9(8 -9 ) IQR 1 ; voted 45 ; ≥7:42(93.3%)**

Item 32: LLMs may help flag internal inconsistencies (e.g., mismatches between abstracts, tables, and main text), but authors and editors must verify accuracy.
**(SC) Median 8(8 -9 ) IQR 1 ; voted 45 ; ≥7:43(95.6%)**

Item 33: Summaries generated by LLMs can assist reviewers in triaging manuscripts, though final evaluations must rely on full human review.
**(NC) Median 8 (6 - 9) IQR 3 ; voted 45 ; ≥7: 31(68.9%)**

Item 34: Editorial workflows may include LLM-based tools for language polishing or reference formatting.
**(C) Median 8(7 -9 ) IQR 2 ; voted 43; ≥7:35(81.4%)**

Item 35: Reviewer comments may be translated or stylistically improved with LLM assistance.
**(NC) Median 8( 5- 9) IQR 4 ; voted 45 ; ≥7:30(66.7%)**

Item 36: Confidential manuscripts should only be processed with secure, journal-approved AI systems; uploading to public tools violates confidentiality obligations.
**(SC) Median 9(8 -9 ) IQR 1 ; voted 44 ; ≥7:40(90.9%)**

Item 37: Journals could allow LLMs to prepare structured summaries of reviewer feedback for authors, provided human editors approve the final version.
**(NC) Median 6( 3- 8) IQR 5 ; voted 44 ; ≥7:20(45.5%)**

Item 38: LLMs may aid in identifying potential reviewers by screening databases for expertise and conflicts of interest.
**(C) Median 8 (7 -9 ) IQR 2 ; voted 44 ; ≥7: 37(84.1%)**

Item 39: Editors and reviewers should disclose any use of LLMs in the assessment process, specifying which LLM was used and for what tasks.
**(C) Median 9(7 -9 ) IQR 2 ; voted 45; ≥7: 39(86.7%)**

Item 40: When LLMs are used for data analysis, manuscript drafting, or interpretation, this involvement should be disclosed in ethics submissions.
**(C) Median 9( 7-9 ) IQR 2 ; voted 44 ; ≥7: 36(81.8%)**

Item 41: Authors must disclose all substantial uses of LLMs in manuscript preparation, specifying how and for what purposes the tools were applied.
**(SC) Median 9 (8 -9 ) IQR 1 ; voted 45 ; ≥7: 44(97.8%)**

Item 42: The acknowledgments section is the appropriate place to disclose the use of LLMs for general support roles such as language editing or formatting assistance.
**(C) Median 9(7 -9 ) IQR 2 ; voted 45 ; ≥7:37(82.2%)**

Item 43: A standardized “AI Disclosure Statement” section, similar to conflict-of-interest declarations, should be added to manuscripts to harmonize reporting.
**(SC) Median 9 (8 -9 ) IQR 1 ; voted 45 ; ≥7: 38(84.4%)**

Item 44: The methods section is the appropriate place to disclose the use of LLMs for research-related tasks such as enhancing search strategies or summarizing literature.
**(NC) Median 8 (6 -9 ) IQR 3 ; voted 44 ; ≥7:31(70.5%)**

Item 45: Cover letters should disclose LLM use to editors, specifying its role even if already mentioned in the manuscript.
**(NC) Median 8 (6 -9 ) IQR 3 ; voted 45 ; ≥7:33(73.3%)**

Item 46: Disclosure statements should specify the tool name, version, access date, and scope of use.
**(SC) Median 9(8 -9 ) IQR 1 ; voted 44 ; ≥7: 41(93.2%)**

Item 47: Journals should provide authors with standardized disclosure templates to ensure uniformity across submissions.
**(SC) Median 9 (8 -9 ) IQR 1 ; voted 45 ; ≥7: 42(93.3%)**

Item 48: Minor uses, such as spelling or punctuation checks on single words or short sentences, do not require disclosure.
**(NC) Median 8 (5.75 -9 ) IQR 3.25 ; voted 44 ; ≥7:31(70.5%)**

Item 49: Failure to disclose significant LLM use should be treated as a serious breach, with grounds for manuscript rejection or retraction.
**(C) Median 8 (7 -9 ) IQR 2 ; voted 44 ; ≥7:40(90.9 %)**

Item 50: Publishers and journals should emphasize that disclosure promotes transparency, trust, and responsible use—not punishment.
**(SC) Median 9 (8 -9 ) IQR 1 ; voted 45 ; ≥7: 43(95.6%)**

Item 51: Authors should archive prompts, parameters, and AI workflows in supplemental materials.
(NC) **Median 7( 5-8 ) IQR 3 ; voted 44 ; ≥7:26(59.1%)**

Item 52: LLMs are prone to generating “hallucinations,” such as fabricated data, references, or plausible but false statements, which could mislead readers.
**(SC) Median 9( 8- 9) IQR 1 ; voted 45 ; ≥7: 41(91.1%)**

Item 53: Over-reliance on LLMs risks eroding core scholarly skills such as critical thinking, originality, and scientific writing.
**(C) Median 9(7 -9 ) IQR 2 ; voted 45 ; ≥7: 39(86.7%)**

Item 54: LLM use may inadvertently expose confidential or unpublished material, risking intellectual property breaches and editorial trust.
**(C) Median 8(7 -9 ) IQR 2 ; voted 45 ; ≥7:39(86.7%)**

Item 55: Because LLMs are trained predominantly on Western, English-language sources, they may perpetuate biases and underrepresent diverse populations.
(NC) **Median 8 (6 -9 ) IQR 3 ; voted 45 ; ≥7:33(73.3%)**

Item 56: Outputs from LLMs are non-deterministic; the same prompt can yield different responses depending on the model version or context, complicating reproducibility.
**(C) Median 8 (7 -9 ) IQR 2 ; voted 44 ; ≥7:34(77.3%)**

Item 57: The proliferation of low-quality or AI-generated manuscripts risks overwhelming peer review and diminishing publication standards.
**(C) Median 8 (7 -9 ) IQR 2 ; voted 45 ; ≥7:37(82.2%)**

Item 58: Reliance on subscription-based LLMs risks widening inequities between well-resourced and low- or middle-income country authors.
**(C) Median 8 (7 - 9) IQR 2; voted 45 ; ≥7:35(77.8%)**

Item 59: Unchecked proliferation of AI-generated manuscripts may result in LLMs being trained on their own synthetic outputs, leading to “model collapse” and degraded scientific quality.
**(C) Median 8 (7 - 9) IQR 2 ; voted 44; ≥7: 35(79.5%)**

Item 60: Because the quality and context of prompts determine reliability, training in “prompt engineering” should be part of responsible academic practice.
(NC) **Median 7(6 -8 ) IQR 2 ; voted 45; ≥7:25(55.6%)**

Item 61:This consensus should be treated as a living document, updated regularly to reflect emerging evidence, technology, and ethical developments.

**(SC) Median 9 (8 -9 ) IQR 1; voted 45 ; ≥7: 42(93.3%)**

**Round 3 – Statements and Voting Results**

SC: Strong consensus; C: Consensus; NC: No Consensus; CA: Consensus against

Item 4: Failure to transparently disclose the use of LLMs in manuscript preparation undermines trust, and deliberate deception regarding such use constitutes academic misconduct

**(SC) Median 9 (8-9) IQR 1; voted 41,≥7 39(95.1%)**

Item 5: It is the responsibility of journals to clarify that LLMs must be acknowledged, with a description of their specific contribution, in the same way as professional language editing or translation services

**(SC) Median 8 (8-9) IQR 1; voted 42,≥7 39(92.9%)**

Item 7: The inclusion of any LLM-generated content is acceptable only when its creation and use are transparently disclosed and compliant with journal guidelines.

**(SC) Median 9 (8-9) IQR 1; voted 40,≥7 36(90.0%)**

Item 8: Permitted, disclosed, and supervised use of LLMs, in line with each journal’s policy, does not diminish the scientific quality or integrity of a manuscript and should not be perceived negatively by authors, peer reviewers, or editors.

**(C) Median 8 (7-9) IQR 2; voted 41,≥7 36(87.8%)**

Item 10:Formatting support, including reference styling, adherence to journal structure, and compliance with word limits, is an appropriate use of LLMs when transparently disclosed.

**(SC) Median 9 (8-9) IQR 1; voted 41,≥7 39(95.1%)**

Item 11:Whenever LLMs are used to summarize a text, the outputs should always be verified against the original sources.

**(SC) Median 9 (8-9) IQR 1; voted 41,≥7 36(87.8%)**

Item 12: It is the author’s responsibility to ensure that layout or caption for figures and tables generated by LLMs are accurate

**(SC) Median 9(9-9) IQR 0; voted 41,≥7 39(95.1%)**

Item 13:LLMs may be used to support conceptual mapping as part of the brainstorming process.

**(C) Median 8 (7-9) IQR 2; voted 41,≥7 32(78.0%)**

Item 14: LLMs may assist in refining search strategies (e.g., suggesting synonyms or Boolean operators) if their use is transparently disclosed.

**(SC) Median 9.5 (8.5-9) IQR 0.5; voted 41; ≥7 39 (95.1%)**

Item 15: Abstracts, titles, or plain-language summaries may be drafted with LLM support, provided all content is reviewed and verified by the authors for accuracy and appropriateness.

**(SC) Median 8(7-9) IQR 2; voted 41,≥7 32(78.0%)**

Item 16: Compliance checks with reporting standards (e.g., CONSORT, PRISMA, STROBE) are an appropriate supportive role for LLMs, provided such use is transparently disclosed, and supplemented by author review.

**(C) Median 8 (7-9) IQR 2; voted 41,≥7 37(90.2%)**

Item 17: Manuscripts translated using automatic tools are acceptable only when their accuracy has been reviewed and confirmed by at least one author or by a professional translator fluent in the target language.

**(C) Median 8 (7-9) IQR 2; voted 41,≥7 37(90.2%)**

Item 18: Journals should consider providing a list of approved LLM tools to allow authors to use them safely without risking confidentiality and to reduce disparities in access.

**(NC) Median 8 (6-9) IQR 3; voted 41,≥7 28(68.3%)**

Item 19: LLMs must not be used to generate primary data or fabricate datasets

**(SC) Median 9 (9-9) IQR 0; voted 41,≥7 38(92.7%)**

Item 25: It is the author’s responsibility to ensure that figures or images created using generative tools are accurate and do not infringe copyright.

**(SC) Median 9(9-9) IQR 0; voted 41,≥7 39(95.1%)**

Item 26: Authors must ensure that any patient-specific recommendations generated by LLMs are accurate

**(SC) Median 9(8-9) IQR 1; voted 41,≥7 34(82.9%)**

Item 27: Peer review reports generated by LLMs are not acceptable, as they breach confidentiality

**(SC) Median 9 (8-9) IQR 1; voted 41,≥7 33(80.5%)**

Item 30: LLMs should not be used as the sole method for identifying submissions with excessive AI-generated content

**(C) Median 8 (7-9) IQR 2; voted 41,≥7 35(85.4%)**

Item 33: Summaries generated by LLMs may assist reviewers in triaging manuscripts, provided the LLM is approved for this task by the journal, and final evaluations remain fully dependent on human review.

**(C) Median 8 (6-9) IQR 3; voted 40,≥7 29(72.7%)**

Item 34: Editorial workflows may include disclosed LLM-based tools for language polishing or reference formatting, provided their use is transparently disclosed

**(C) Median 8 (7-9) IQR 2; voted 40,≥7 36(90.0%)**

Item 35: Reviewers may use LLMs to improve the clarity or language of their feedback; however, they remain fully responsible for the content and integrity of their report.

**(C) Median 8 (7-9) IQR 2; voted 40,≥7 34(85.0%)**

Item 37: Journals could allow LLMs to generate structured summaries of reviewer feedback for authors, provided this use is disclosed and the final summary is approved and verified by humans against the original reviews.

**(NC) Median 7 (5.5-9) IQR 3.5; voted 39,≥7 27(69.2%)**

Item 38: LLMs may aid in identifying potential reviewers by screening databases for expertise and conflicts of interest, however, editors must retain ultimate responsibility for reviewer assignment.

**(C) Median 9 (7-9) IQR 2; voted 40,≥7 37(92.5%)**

Item 39: Editors and reviewers should disclose any use of LLMs in the assessment process, specifying which LLM was used and the acceptable tasks it supported.

**(SC) Median 8 (7.5-9) IQR 1.5; voted 39,≥7 34(87.2%)**

Item 40:Prior to using LLMs to analyze patient data, authors must obtain approval from an institutional ethics committee to ensure patient privacy and compliance with applicable regulations.

**(C) Median 9 (7-9) IQR 2; voted 41,≥7 34(82.9%)**

Item 42: The acknowledgments section is the appropriate place to disclose the use of LLMs for general support roles only such as language editing or formatting assistance.

**(NC) Median 8 (5-9) IQR 4; voted 41,≥7 29(70.7%)**

Item 44: Any use of LLMs related to the research methodology should be transparently reported in the Methods section.

**(SC) Median 9(8-9) IQR 1; voted 41,≥7 40(97.6%)**

Item 45: Authors are encouraged to disclose the use of LLMs in the cover letter, indicating the nature, extent, and purpose of their contribution.

**(C) Median 8 (7-9) IQR 2; voted 41,≥7 35(85.4%)**

Item 48:Single words or short sentences spelling or punctuation checks, do not require disclosure, similar to conventional grammar software

**(C) Median 8 (7-9) IQR 2; voted 41,≥7 31(75.6%)**

Item 49: Any prohibited use of LLMs should be treated as a serious breach, with grounds for manuscript rejection or retraction.

**(SC) Median 9 (8-9) IQR 1; voted 41,≥7 38(92.7%)**

Item 51: Authors are encouraged to document LLM prompts, parameters, and workflows for transparency and accountability.

**(C) Median 8 (7-9) IQR 2; voted 40,≥7 35(87.5%)**

Item 53: Over-reliance on LLMs without adequate training may erode essential scholarly skills, including critical thinking, originality, and scientific writing.

**(SC) Median 9(8-9) IQR 1; voted 41,≥7 36(87.8%)**

Item 54: LLM use may inadvertently expose confidential or unpublished material, risking intellectual property breaches

**(SC) Median 9 (8-9) IQR 1; voted 41,≥7 37(90.2%)**

Item 55: Preference should be given to LLMs developed from diverse, multilingual datasets rather than those trained solely on Western or English-language sources, to minimize bias and improve inclusivity.

**(NC) Median 7(6-9) IQR 3; voted 40,≥7 26(65.0%)**

Item 56: Outputs from LLMs are not predictable; the same prompt can yield different responses depending on the model version or context, complicating reproducibility.

**(C) Median 8(7-9) IQR 2; voted 41,≥7 34(82.9%)**

Item 57: The proliferation of low-quality AI-generated manuscripts risks overwhelming peer review and diminishing publication standards.

**(SC) Median 9(8-9) IQR 1; voted 41,≥7 34(82.9%)**

Item 58: Reliance on updated, subscription-based LLMs versus older, freely accessible models risks widening inequities between well-resourced and low- or middle-income country authors.

**(NC) Median 8 (6-9) IQR 3; voted 41,≥7 30(73.2%)**

Item 59: Uncontrolled growth of AI-generated manuscripts could make future AI tools train on their own artificial content, which may cause “model collapse" lowering the quality of scientific output.

**(C) Median 8 (7-9) IQR 2; voted 39,≥7 31(79.5%)**

Item 60: Training in the use of LLMs should be considered part of responsible academic practice.

**(SC) Median 9 (7-9) IQR 2; voted 41,≥7 37(90.2%)**

Supp 1 :Publishers and editors, share responsibility for implementing transparent policies regarding LLM use

**(SC) Median 9 (8-9) IQR 1; voted 41,≥7 40(97.6%)**

Supp 2 :Authors should not request LLMs to modify or rephrase copyrighted material without the explicit consent of the copyright holder.

**(SC) Median 9 (7-9) IQR 2; voted 41,≥7 34(82.9%)**

Supp 3 : The original source must always be properly cited, even when permission to use or adapt the material has been obtained.

**(SC) Median 9(8-9) IQR 1; voted 41,≥7 37(90.2%)**
**Table illustrating the evaluation of each item over the rounds**

| **#Item** | **Round 2** | **Round 3** |
| --- | --- | --- |
| **Item 1** | SC; accepted |  |
| **Item 2** | SC; accepted |  |
| **Item 3** | SC; accepted |  |
| **Item 4** | C; rephrased | SC; accepted |
| **Item 5** | C; rephrased | SC; accepted |
| **Item 6** | SC; accepted |  |
| **Item 7** | C; rephrased | SC; accepted |
| **Item 8** | NC; reformulated | C;accepted |
| **Item 9** | SC; accepted |  |
| **Item 10** | C; rephrased | SC; accepted |
| **Item 11** | NC; reformulated | SC; accepted |
| **Item 12** | NC; reformulated | SC; accepted |
| **Item 13** | NC; reformulated | C; accepted |
| **Item 14** | C; rephrased | SC; accepted |
| **Item 15** | NC; reformulated | SC; accepted |
| **Item 16** | C; rephrased | C; accepted |
| **Item 17** | C; rephrased | C; accepted |
| **Item 18** | NC; reformulated | NC; rejected |
| **Item 19** | C; rephrased | SC; accepted |
| **Item 20** | SC; accepted |  |
| **Item 21** | SC; accepted |  |
| **Item 22** | SC; accepted |  |
| **Item 23** | SC; accepted |  |
| **Item 24** | SC; accepted |  |
| **Item 25** | C; rephrased | SC; accepted |
| **Item 26** | C; rephrased | SC; accepted |
| **Item 27** | C; rephrased | SC; accepted |
| **Item 28** | SC; accepted |  |
| **Item 29** | SC; accepted |  |
| **Item 30** | NC; reformulated | C; accepted |
| **Item 31** | SC; accepted |  |
| **Item 32** | SC; accepted |  |
| **Item 33** | NC; reformulated | C; accepted |
| **Item 34** | C; rephrased | C; accepted |
| **Item 35** | NC; reformulated | C; accepted |
| **Item 36** | SC; accepted |  |
| **Item 37** | NC; reformulated | NC; rejected |
| **Item 38** | C; rephrased | C; accepted |
| **Item 39** | C; rephrased | SC; accepted |
| **Item 40** | C; rephrased | C; accepted |
| **Item 41** | SC; accepted |  |
| **Item 42** | C; rephrased | NC; rejected |
| **Item 43** | SC; accepted |  |
| **Item 44** | NC; reformulated | SC; accepted |
| **Item 45** | NC; reformulated | C; accepted |
| **Item 46** | SC; accepted |  |
| **Item 47** | SC; accepted |  |
| **Item 48** | NC; reformulated | C; accepted |
| **Item 49** | C; rephrased | SC; accepted |
| **Item 50** | SC; accepted |  |
| **Item 51** | NC; reformulated | C; accepted |
| **Item 52** | SC; accepted |  |
| **Item 53** | C; rephrased | SC; accepted |
| **Item 54** | C; rephrased | SC; accepted |
| **Item 55** | NC; reformulated | NC; rejected |
| **Item 56** | C; rephrased | C; accepted |
| **Item 57** | C; rephrased | SC; accepted |
| **Item 58** | C; rephrased | NC; rejected |
| **Item 59** | C; rephrased | C; accepted |
| **Item 60** | NC; reformulated | SC; accepted |
| **Item 61** | SC; accepted |  |
| **Supplementary Item 1** | – | SC; accepted |
| **Supplementary Item 2** | – | SC; accepted |
| **Supplementary Item 3** | – | SC; accepted |

**CREDES checklist**

**1. Rationale for the choice of the Delphi technique**

“All members of the scientific ecosystem—authors, reviewers, editors, and publishers—have encountered this novel tool, and face the urgent need to identify its possible applications, benefits, limitations, and the ethical implications associated with its use.Given the growing ubiquity of these tools in academic workflows, and the need to balance the concerns associated with their use and the potential benefits of responsible application, the present manuscript reports a Delphi consensus” and “The Delphi method was selected because it enables a structured and iterative collection of expert opinions in areas where empirical data are limited and policy development relies on informed judgment. A consensus-based approach is considered the most appropriate to capture diverse expert perspectives while minimizing the influence of dominant individuals and reducing potential geographic or institutional bias.”

**2. Planning and design**

“The study comprised three mandatory rounds (one qualitative and two quantitative rating rounds), with an optional fourth round for validation. All procedures were conducted online using LimeSurvey (LimeSurvey GmbH, Hamburg, Germany).”

**Method section**

**3. Definition of consensus**

“Consensus thresholds were defined a priori as follows: strong consensus (median ≥ 8, IQR ≤ 1.5, and ≥ 80% of participants rating 7–9); consensus (median ≥ 7, IQR ≤ 2.0, and ≥ 70% rating 7–9); and consensus against (median ≤ 3, IQR ≤ 2.0, and ≥ 70% rating 1–3). Items not meeting these criteria were classified as showing no consensus.”

**4. Informational input**

Supplementary Material 1 and 2

**5. Prevention of bias**

**“**These draft statements were further reviewed by a third independent reviewer (MFP) who was blinded” and “All responses were anonymised prior to analysis. Identifiers such as journal name or participant name were stored separately and were accessible only to the project administrators “

**6. Interpretation and processing of results**

### “The RULE-AP Delphi project was approved by the Clinical Research Ethics Committee of Ondokuz Mayis University (approval number 2025/456). Informed consent was obtained from each participant through explicit checkbox confirmation at the beginning of the first-round survey, following a detailed explanation of the project’s aims and objectives. The protocol and the data from each round were available for consultation for the working group at Open Science Framework (reference: osf.it/973vf, first registered on: August 28, 2025)

### All responses were anonymised prior to analysis. Identifiers such as journal name or participant name were stored separately and were accessible only to the project administrators (ADC and BD). Quantitative data were analyzed descriptively using medians, IQRs, and percentage distributions.

The sample size was determined *a priori*. The study was designed to proceed only if at least 20% of eligible journals agreed to participate. Given that 147 journals met the inclusion criteria, the minimum required number of consenting editors was set at 29.”

**7. External validation (if applicable)**

Not applicable

### **Reporting-Focused Items**

**8. Purpose and rationale (reporting)**

“The Delphi method was selected because it enables a structured and iterative collection of expert opinions in areas where empirical data are limited and policy development relies on informed judgment. A consensus-based approach is considered the most appropriate to capture diverse expert perspectives while minimizing the influence of dominant individuals and reducing potential geographic or institutional bias.”

**9. Expert panel**

A total of 147 journals were identified as eligible for inclusion (S1). Following the initial invitation and subsequent reminders, 53 EICs from 53 journals (36.0%) agreed to participate in the project—one journal was represented by two EICs, and two journals shared the same EIC. Two EICs from two journals (1.4%) declined participation, and no feedback was received from the remaining 92 journals (62.5%). Among the 53 participating EICs, eight delegated a member of their editorial board to take part in the project on their behalf (see S1 for a full list of the included members and their corresponding roles).

The workgroup’s journals were:*A & A Practice, Acta Anaesthesiologica Belgica, Agri, Anaesthesia and Intensive Care, Anaesthesia Critical Care & Pain Medicine, Anaesthesiology Intensive Therapy, Anesthesia and Pain Medicine, Anasthesiologie und intensivmedizin, Anestezi Dergisi, Anesteziologie a Intenzivni Medicina, Anesthesiology, Annals of Critical Care, Best Practice & Research-Clinical Anaesthesiology, Brazilian Journal of Anesthesiology, British Journal of Anaesthesia, Canadian Journal of Anesthesia-Journal canadien d anesthesie, Canadian Journal of Pain, Clinical Journal of Pain, Colombian Journal of Anesthesiology, Critical Care and Resuscitation, Current Opinion in Anesthesiology, Douleur et Analgesie, Indian Journal of Anaesthesia, Indian Journal of Clinical Anaesthesia, Indian Journal of Respiratory Care, International Journal of Obstetric Anesthesia, Journal of Anesthesia Analgesia and Critical Care, Journal of Cardiothoracic and Vascular Anesthesia, Journal of Clinical Anesthesia, Journal of Critical Care Medicine, Journal of Opioid Management, Journal of Pain, Journal of Pain Research, Journal of Palliative Medicine, Korean Journal of Anesthesiology, Minerva Anestesiologica, Open Pain Journal, Pain Research and Management, Palliative Medicine, Palliative Medicine Reports, Patient Safety in Surgery, Pediatric Anesthesia and Critical Care Journal, Perioperative Care and Operating Room Management, Regional Anesthesia and Pain Medicine, Revista Espanola de Anestesiologia y Reanimacion, Saudi Journal of Anaesthesia, Scandinavian Journal of Pain, Seminars in Arthritis and Rheumatism, Sri Lankan Journal of Anaesthesiology, The Journal of Headache and Pain, Trends in Anaesthesia and Critical Care, Turkish Journal of Anaesthesiology and Reanimation and Update in Anaesthesia.*

Most of the workgroup’s journals publish exclusively in English, but some accept or publish in a bilingual format (including Czech, French, Portuguese, Russian, Slovak, Spanish, and Turkish). The workgroup is composed of members from all continents, with a predominance of North America (19) and Europe (18), followed by Asia (6), Oceania (3), South America (3), and Africa (1).

**10. Description of methods**

See Methods (in particular Modified Delphi Process paragraph)

**11. Procedure**

Supplementary material 1 and 2

**12. Definition and attainment of consensus**

“Consensus thresholds were defined a priori as follows: strong consensus (median ≥ 8, IQR ≤ 1.5, and ≥ 80% of participants rating 7–9); consensus (median ≥ 7, IQR ≤ 2.0, and ≥ 70% rating 7–9); and consensus against (median ≤ 3, IQR ≤ 2.0, and ≥ 70% rating 1–3). Items not meeting these criteria were classified as showing no consensus.”

**13. Results**

**See results + Supplementary Material 1 and 2**

**14. Discussion of limitations**

This Delphi consensus reflects the perspectives of EICs and their delegates from anaesthesiology and pain medicine journals and does not include experts in AI, ethics, or broader scientific publishing. Consequently, the identified risks and recommended practices should be interpreted as the viewpoint of editors managing LLMs in their daily editorial workflows rather than as definitive evidence-based recommendations. The participation rate (36.0% of eligible journals) may introduce selection bias, and while the Delphi method facilitates structured consensus, it remains inherently subjective and dependent on the expertise and experiences of participants. Finally, the rapidly evolving nature of LLM technology means that guidance will likely need regular updating to remain relevant and aligned with emerging evidence and ethical standards.

**15. Adequacy of conclusions**

The RULE-AP Delphi project provides editor-informed guidance for the responsible use of LLMs in manuscript preparation, peer review, and editorial workflows. Key principles include transparency, human accountability, and human verification of all tasks and outputs. This guidance reflects current knowledge but may quickly be superseded as technology and understanding evolve. Additional caution is recommended for areas lacking strong consensus.

**16. Publication and dissemination plans**

In a peer-reviewed journal
